# Robust Radiomic Signatures of Intervertebral Disc Degeneration From MRI

**DOI:** 10.1097/BRS.0000000000005435

**Published:** 2025-06-20

**Authors:** Terence McSweeney, Aleksei Tiulpin, Narasimharao Kowlagi, Juhani Määttä, Jaro Karppinen, Simo Saarakkala

**Affiliations:** a Research Unit of Health Sciences and Technology, University of Oulu, Oulu, Finland; b Neurocenter Oulu, Oulu University Hospital, Oulu, Finland; c Wellbeing Services County of South Karelia, Lappeenranta, Finland; d Department of Diagnostic Radiology, University Oulu Hospital, Oulu, Finland

**Keywords:** radiomics, texture analysis, deep learning, intervertebral disc degeneration, lumbar spine, low back pain, magnetic resonance imaging

## Abstract

**Study Design.:**

A retrospective analysis.

**Objective.:**

The aim of this study was to identify a robust radiomic signature from deep learning segmentations for intervertebral disc (IVD) degeneration classification.

**Summary of Data.:**

Low back pain (LBP) is the most common musculoskeletal symptom worldwide and IVD degeneration is an important contributing factor. To improve the quantitative phenotyping of IVD degeneration from T2-weighted magnetic resonance imaging (MRI) and better understand its relationship with LBP, multiple shape and intensity features have been investigated. IVD radiomics has been less studied but could reveal subvisual imaging characteristics of IVD degeneration.

**Materials and Methods.:**

We used data from Northern Finland Birth Cohort 1966 members who underwent lumbar spine T2-weighted MRI scans at age 45 to 47 (n=1397). We used a deep learning model to segment the lumbar spine IVDs and extracted 737 radiomic features, as well as calculating IVD height index and peak signal intensity difference. Intraclass correlation coefficients across image and mask perturbations were calculated to identify robust features. Sparse partial least squares discriminant analysis was used to train a Pfirrmann grade classification model.

**Results.:**

The radiomics model had balanced accuracy of 76.7% (73.1%–80.3%) and Cohen’s kappa of 0.70 (0.67–0.74), compared with 66.0% (62.0%–69.9%) and 0.55 (0.51–0.59) for an IVD height index and peak signal intensity model. 2D sphericity and interquartile range emerged as radiomics-based features that were robust and highly correlated to Pfirrmann grade (Spearman’s correlation coefficients of −0.72 and −0.77, respectively).

**Conclusion.:**

Based on our findings, these radiomic signatures could serve as alternatives to the conventional indices, representing a significant advance in the automated quantitative phenotyping of IVD degeneration from standard-of-care MRI.

Low back pain (LBP) is the number one disease accounting for years lived with disability globally^1^ and carries a huge disease burden.^2^ Intervertebral disc (IVD) degeneration is considered an important contributing factor to LBP^3–6^ but is also highly prevalent in asymptomatic populations.^7^ The specific role of IVD degeneration in LBP may be obscured by the subjective and coarse nature of qualitative IVD degeneration grading from clinical magnetic resonance imaging (MRI). State-of-the-art deep learning (DL) and radiomics-based approaches offer a means to overcome this by extracting more quantitative, granular, and discriminative information from MRI of the IVD, which could facilitate novel insights into the mechanisms of IVD degeneration and its contribution to LBP.

The current approach to degeneration grading relies on visual evaluation using semi-quantitative tools such as Pfirrmann’s five-point scale, which is based on gross structural changes of the IVD^8^ (Figure 1). This method is subjective, inherently costly of time and expertise, and has variable intra-rater and inter-rater reliability.^9–12^ Deep learning (DL) models have been trained to automatically classify Pfirrmann grade, but the lack of interpretability and bias to training data means their utility is also limited.^13,14^

**Figure 1:**
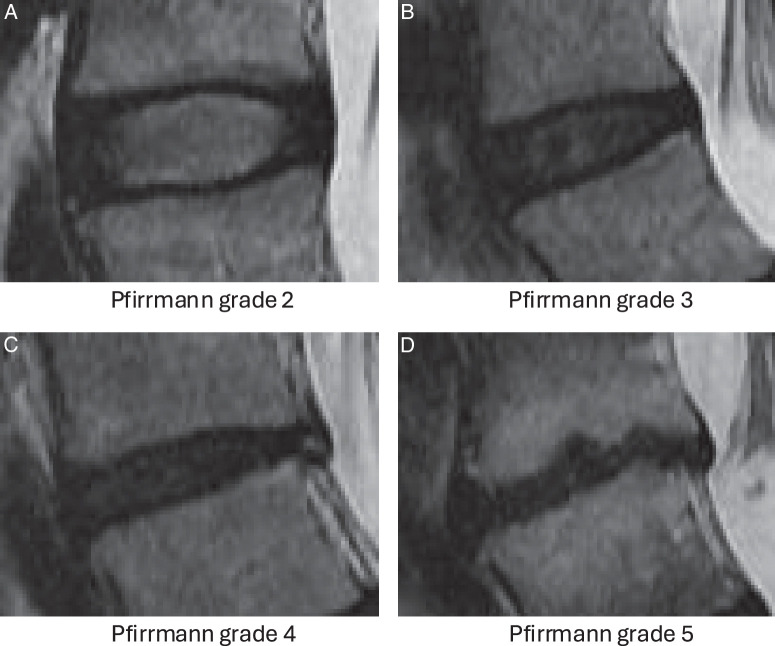
Examples of Pfirrmann grade 2 to 5 IVD degeneration. (A) Pfirrmann grade 2 has a hyper-intense but somewhat inhomogeneous nucleus pulposis (NP), clear distinction between the NP and annulus fibrosis (AF), and normal IVD height. (B) Pfirrmann grade 3 has an inhomogeneous gray NP with intermediate intensity, unclear distinction between the NP and AF, and a slight decrease in IVD height. (C) Pfirrmann grade 4 has a hypo-intense and inhomogeneous gray to black NP, loss of distinction between the NP and AF, and moderate decrease in IVD height. (D) Pfirrmann grade 5 has a hypointense inhomogeneous NP, loss of distinction between the NP and AF, and collapsed IVD space.

Features derived from quantitative MRI have shown potential to identify the earliest stages of IVD degeneration,^15^ but their use is exploratory and they require further validation before widespread implementation.^16^ From the most common clinical sequence, T2-weighted MRI, measures of IVD height^17–20^ and peak signal intensity difference^20,21^ are well established and have been considered candidate imaging biomarkers of IVD degeneration.^22^ However, these represent only a fraction of the possible shape, intensity, and texture features that can be extracted from T2-weighted MRI of the IVD, collectively referred to as radiomics. The radiomic hypothesis is that a given tissue’s phenotypic, proteomic, and genomic information can be inferred using high-throughput analysis of macroscopic imaging features.^23^ In the case of the IVD, radiomics may reveal subtle subvisual changes relevant to the objective phenotyping of IVD degeneration and measurement of its severity.

Despite an increasing application to musculoskeletal imaging challenges, relatively few studies have investigated IVD radiomics from clinical MRI. Most studies have tested individual shape or intensity features^18,24–29^ although some have used larger sets of texture features.^30–32^ IVD radiomics from clinical MRI have also been investigated for the classification of painful annular fissures,^33^ spine surgery prediction,^34^ and LBP classification.^35–37^ Most studies have relied on manual or semi-automated segmentation, have small sample sizes, and the robustness of the features is not reported or untested. As such, it is still unknown whether IVD radiomics can deliver on their purported potential to “capture additional information not currently used”^23^ and maximize the value of information in standard-of-care lumbar spine MRI. This is particularly relevant to clinical sequences, which are widely available but whose rich data may be underutilized for quantifying IVD degeneration.

The aim of this study was to identify a robust radiomic signature for IVD degeneration using DL segmentation in a population-based cohort. To test this, we applied perturbations to DL segmentations before radiomic feature extraction. The radiomic features and conventional indices were then compared for Pfirrmann grade classification accuracy and reliability. We also aimed to examine the features of highest relevance in the resulting model to test for additional information not captured by the conventional indices.

## MATERIALS AND METHODS

### Data

#### Population

The study population is drawn from the Northern Finland Birth Cohort 1966 (NFBC1966), which is a prospective birth cohort started in 1965. The follow-up and MRI data used in this study were collected from 2012 to 2014 when participants were between 45 and 47 years of age.^38,39^ Ethics approval for the NFBC1966 was granted by the Northern Ostrobothnia Hospital District Ethics Committee. The population demographics are detailed in Table 1, and further information regarding the recruitment and population characteristics is available in Nordström *et al.* ^38^

**TABLE 1:** Population Characteristics and Degeneration Prevalence

	Female, N (%)/*M*±SD	Male, N (%)/*M*±SD	Total, N (%)/*M*±SD
Participants	745 (53.3)	652 (46.7)	1397 (100)
Height (cm)	164.8±5.9	178.6±6.2	171.2±9.2
Weight (kg)	71.6±14.3	86.2±12.8	78.4±15.4
BMI (kg/m^2^)	26.4±5.2	27.0±3.7	26.7±4.6
Smoking pack-years			
0	616 (82.7)	514 (78.5)	1130 (80.9)
1–10	58 (7.8)	53 (8.1)	111 (8.0)
11–50	68 (9.1)	74 (11.4)	142 (10.2)
>50	3 (0.4)	11 (1.7)	14 (1.0)
LBP during the last 12 mo			
None	307 (41.2)	282 (43.3)	589 (42.2)
1–7 d	112 (15.0)	108 (16.6)	220 (15.8)
8–30 d	143 (19.2)	114 (17.5)	257 (18.4)
>30 d	129 (17.3)	106 (16.3)	235 (16.8)
Daily	54 (7.2)	42 (6.4)	96 (6.9)
Pfirrmann grade			
Grade 2	1860 (50.1)	1381 (42.6)	3241 (46.6)
Grade 3	1188 (32.0)	1017 (31.3)	2205 (31.7)
Grade 4	503 (13.6)	641 (19.8)	1144 (16.4)
Grade 5	159 (4.3)	206 (6.4)	365 (5.3)

BMI indicates body mass index; LBP, low back pain; *M*, mean; SD, standard deviation

#### Magnetic Resonance Imaging

The study used T2-weighted sagittal MRI acquired on 1.5-Tesla equipment (Signa HDxt; General Electric, Milwaukee, WI). Sagittal T2-weighted fast-recovery fast spin-echo images were captured with repetition time/effective echo time 3500/112 ms, 4 averages, 280×280 mm field of view, 512×512 slice dimensions, 3 mm slice thickness, and 1 mm interslice gap. Images unsuitable for radiomic feature calculation due to metal artefacts (n=3), developmental anomaly (n=1), severe scoliosis (n=1), and poor image quality or missing sequences (n=24), were excluded.

The study sample was limited to those participants for whom consensus IVD degeneration evaluations were available.^9^ Given the low prevalence of Pfirrmann grade 1 at the 46-year follow-up, Pfirrmann grades 1 and 2 have historically been pooled, resulting in 4 grades (2–5) available for analysis.

The final data set consisted of 6985 IVDs from 1397 participants. The prevalence and distribution of degenerative changes is shown in Table 1. Data were split into a development set (80%, n=5588 IVDs) and test set (20%, n=1397 IVDs) stratified by participant and Pfirrmann grade.

#### Segmentation

We used an existing DL model^40^ trained on the NFBC1966 to segment the lumbar spine IVDs from 4/5 sagittal slices centred on the numerical mid-sagittal slice/s. The DL model performance was evaluated against a set of 300 IVDs and adjacent vertebral bodies from the development set, which were manually annotated by an experienced musculoskeletal researcher (JM). Jaccard index and 95% Hausdorff distance were used to assess the quality of the DL segmentation model. The DL segmentations were screened to remove slices where the DL segmentation failed completely.

#### Image-mask Perturbations

**Figure 2:**
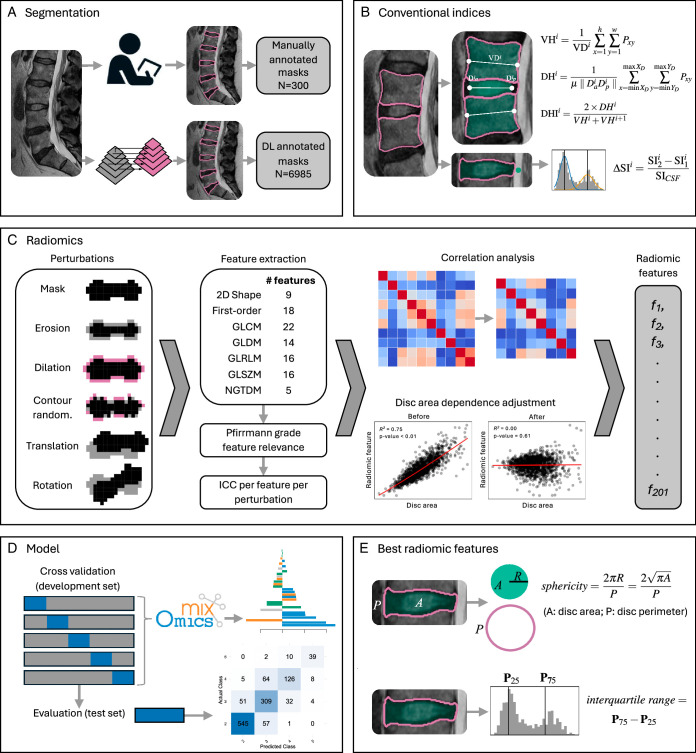
Overview of the methodological steps taken in the study. (A) Deep learning and manual segmentation of the IVD and vertebral body. (B) Calculation of the conventional indices per IVD. (C) Radiomic feature calculation and robustness analysis. (D) Classification model training and testing. (E) Calculation of the two best-performing radiomic features.

Additional sets of perturbed images and DL masks were generated for the set of 300 manually annotated IVDs in the development set. The settings for the perturbations are detailed in Table 2, with examples of the mask perturbations illustrated schematically in Figure 2C.

**TABLE 2:** Image and Mask Perturbation Settings

Perturbation	Settings	Application
Dilation	2 × 2 pixel square dilation kernel	Mask
Erosion	2 × 2 pixel square erosion kernel	Mask
Contour randomization	Random application of 2 × 2 pixel square erosion and dilation kernels to the whole mask contour	Mask
Translation	Random positive and negative translation of between 1 and 10 pixels along the *x* and *y* axes using affine transform	Image + mask
Rotation	Random rotation of between 1 and 360 degrees using affine transform	Image + mask
Random Gaussian noise	Random values from Gaussian distribution of mean 0 and standard deviation 8 added to the image. New image values clipped between 0 and 255	Image

All transforms and contour perturbations were applied using OpenCV in Python 3.9.

### Radiomics

Features were calculated for all IVDs segmented by the DL model in the development and test datasets using the PyRadiomics package^41^ and following the Imaging Biomarkers Initiative standards.^42^ Due to the anisotropy of T2-weighted lumbar spine sequences, all features were calculated in 2D per slice and mean aggregated. Images were normalized, discretised to a bin width of 16 and resampled twofold. The original (preprocessed) images were used, along with Laplacian of Gaussian filtered and wavelet transformed images. From each set of images, 18 first-order intensity-based, 22 gray level co-occurrence (GLCM), 14 gray level dependence matrix (GLDM), 16 gray level run length matrix (GLRLM), 16 gray level size zone matrix (GLSZM), and 5 neighboring gray tone difference matrix (NGTDM) features were calculated. From the masks, 9 shape features were also calculated, resulting in a final set of 737 features per IVD. The same features were extracted from the 300 manually annotated IVDs and the 9 matched sets of image-mask perturbations.

#### Feature Relevance, Robustness, and Correlation Analysis

Features of low relevance to Pfirrmann grade (Spearman’s correlation coefficient *P*-value >0.05) were removed before carrying out the robustness analysis. The intraclass correlation coefficient (ICC) for each feature for each perturbation was then calculated against the manual segmentations. Hierarchical clustering implemented in Scikit-learn^43^ was used to select the cluster of features with the highest ICC values across perturbations. Pairwise feature dependence was identified using Spearman’s correlation coefficient >0.90 and the feature with lower variance in each pair of highly correlated features was removed.

#### IVD Surface-area Dependence Adjustment

Several radiomic features are area dependent and correcting for this has been shown to improve the performance of radiomics models.^44,45^ We tested the IVD features for their correlation to IVD 2D surface area in the development set and then used the trained models to adjust the features in both the development and test sets.

#### Conventional Indices

IVD height index was calculated using an area-based method^17,18^ (Figure 2B). Peak signal intensity difference was calculated using the method of Waldenberg *et al.* ^21^ with the addition of a normalization step (Figure 2B). Peak signal intensity difference was normalized using the mean cerebrospinal fluid signal intensity from a manually placed circular region of interest (ROI) of 3 mm diameter placed posterior to each IVD. The indices were calculated across 4/5 mid-sagittal slices and mean-aggregated. The ICCs for the conventional indices from manual *versus* DL segmentation were calculated in the same manner as the radiomic feature ICCs.

### Model Training and Evaluation

A sparse partial least squares discriminant analysis (sPLSDA) model implemented using the R package mixOmics^46^ was trained on the development set to classify the IVDs for Pfirrmann grade. Repeated 5-fold cross-validation was used to evaluate the model for the optimum number of components and feature sparsity per component based on balanced error rate. The final model was used to predict Pfirrmann grade in the test set. A second sPLSDA model was trained and tested, excluding shape and first-order features, to examine the performance of texture features alone.

Feature importance was evaluated based on the sPLSDA loadings and Spearman’s correlation coefficient for Pfirrmann grade. Feature stability was evaluated as the proportion of cross-validation folds (across repeats) where a feature was selected for a given component.

A support vector machine (SVM) was trained to classify Pfirrmann grade using IVD height index and peak signal intensity difference and evaluated on the test set. SVM hyperparameters were tuned using repeated 5-fold cross-validation in the development set.

Further details of the radiomic and statistical methods are available in the supplementary material, Supplemental Digital Content 1, http://links.lww.com/BRS/C751 and the analysis scripts are available online at github.com/termcs/radiospineomics.

## RESULTS

### Data

#### Segmentation Reliability

Manual screening of the DL mask inferences resulted in the removal of lateral slices where the DL model failed. The most common reason for this was the presence of scoliosis, where the central region of the IVD or vertebral bodies was not captured by the numerical mid-sagittal slices. In all, 5.6% of IVDs had one to three slices removed for this reason, but in all cases, at least one accurately segmented mask per IVD could be retained.

Out of 300 manually annotated IVDs, the DL model misidentified the level in 17 cases. Excluding these, average agreement between the annotator (J.M.) and the DL segmentations was Dice coefficient 0.90 (0.87–0.94), Jaccard index 0.82 (0.77–0.88), and 95% Hausdorff of 2.12 mm (0.60–3.65 mm).

#### Feature Relevance, Reliability, and Correlation Analysis

Three hundred and seven features were removed because of low relevance to the Pfirrmann grade. Clustering of the remaining features resulted in a set of 280 features with the highest ICC values across perturbations. Seventy-nine highly correlated features were then removed, resulting in a final set of 201 features. Out of the 201 radiomic features retained, ICC ranged from 0.63 to 0.99, with 125 features having an ICC >0.90.

ICC values and 95% confidence intervals for IVD height index and peak signal intensity difference were 0.81 (0.49–0.91) and 0.60 (0.22–0.78), respectively.

#### IVD Surface-area Dependence Adjustment

Twenty-seven features were identified as highly correlated to IVD area and were adjusted in the development and test sets using the coefficients of the model from the development set.

### Radiomic Signature

#### Classification Model Performance

The classification performance of the SVM model using conventional indices on the test set was balanced accuracy (BA) of 66.0% (62.0%–69.9%) and Cohen’s kappa of 0.55 (0.51–0.59). Repeated cross-validation resulted in a 24-component sPLSDA model using the texture features alone that had BA of 72.5% (68.8%–76.1%) and Cohen’s kappa of 0.66 (0.62–0.69) in the test set and a 23-component sPLSDA model using all radiomics features that had BA of 76.8% (73.1%–80.3%) and Cohen’s kappa of 0.71 (0.67–0.74) in the test set. The full list of features used, their loadings, and stability is available in the Supplementary Material, Supplemental Digital Content 1, http://links.lww.com/BRS/C751.

Given the high importance of 2D sphericity and interquartile range in the full radiomics model, a further SVM model was trained and tested using these two features. This model classified the test set IVDs with a BA of 76.7% (73.1%–80.1%) and Cohen’s kappa of 0.67 (0.64–0.71). Evaluation metrics for all four models are shown in Table 3.

**TABLE 3:** Pfirrmann Grade Classification Model Performance

Metric	Conventional indices, SVM	Best 2 radiomic features, SVM	Texture features, sPLSDA	All features, sPLSDA
Accuracy (%)	71.8 (69.3–74.4)	79.1 (76.7–81.3)	78.7 (76.2–80.9)	**81.4** (**79.3–83.5)**
BA (%)	66.0 (62.0–69.9)	76.7 (73.1–80.1)	72.5 (68.8–76.1)	**76.8** (**73.1–80.3)**
Macro-avg. F1	65.6 (61.8–69.2)	76.0 (72.8–79.2)	0.73 (0.70–0.76)	**0.77** (**0.74–0.80)**
Macro-avg. AUC	0.89 (0.88–0.90)	**0.94** (**0.93–0.95)**	0.91 (0.90–0.92)	0.93 (0.91–0.94)
Cohen’s kappa	0.55 (0.51–0.59)	0.67 (0.64–0.71)	0.66 (0.62–0.69)	**0.71** (**0.67–0.74)**
QW kappa	0.76 (0.73–0.79)	0.84 (0.82–0.87)	0.83 (0.80–0.85)	**0.85** (**0.83–0.87)**
Lin’s CCC	0.76 (0.74–0.78)	0.84 (0.83–0.86)	0.83 (0.81–0.84)	**0.85** (**0.84–0.87)**

Performance metrics in the test set with bootstrapped 95% confidence intervals across implemented models. The highest values per metric are in bold.

AUC indicates area under receiver operator characteristic curve; BA, balanced accuracy; CCC, concordance correlation coefficient; QW, quadratic weighted; sPLSDA, sparse partial least squares discriminant analysis; SVM, support vector machine.

**Figure 3:**
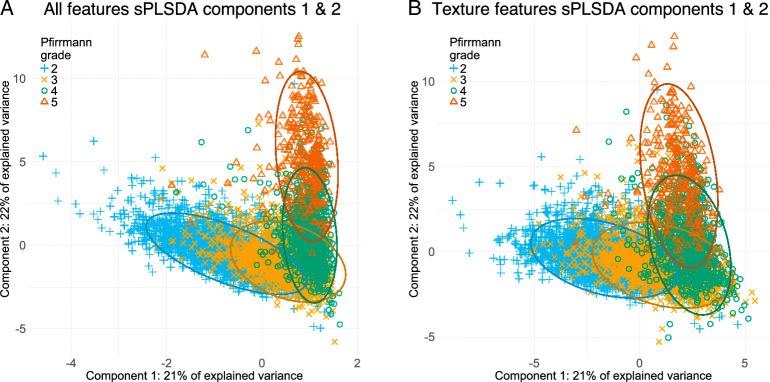
Scatter plots of the first two latent variables of the sPLSDA using all radiomic features (A) and the sPLSDA using texture features only (B). Ellipses represent 95% confidence for each Pfirrmann grade.

**Figure 4:**
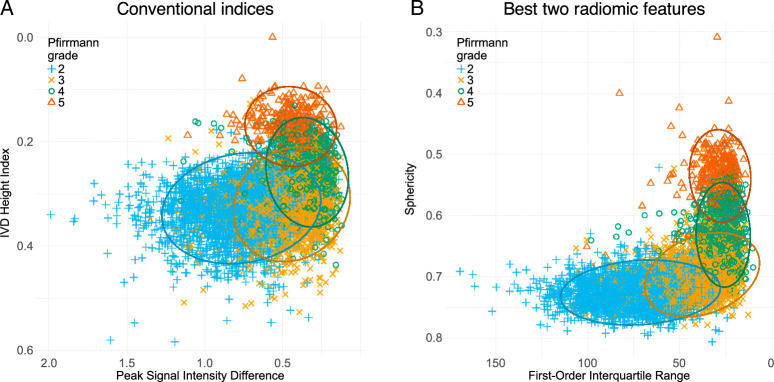
Scatter plots of the two conventional indices (A) and the two radiomic features of highest importance from the sPLSDA model (B). Ellipses represent 95% confidence for each Pfirrmann grade.

Projections of the individual IVDs from the development set onto the first and second latent variables of the sPLSDA for the full radiomics model and the texture features model are shown in Figure 3A and B. Scatter plots of the individual IVDs using the two conventional indices and the top-two radiomic features are shown in Figure 4A and B.

The eight features contributing to the first two components of the full radiomics model are listed alongside the conventional indices in Table 4. Pfirrmann grade Spearman’s correlation coefficient was −0.67 (−0.65, −0.68) for peak signal intensity difference and −0.45 (−0.43, −0.47) for IVD height index, while ICC for these features from DL mask *versus* manual annotated mask was 0.60 and 0.81, respectively. Of the radiomic features with high importance, Spearman’s correlation coefficient was −0.77 (−0.76, −0.78) for interquartile range, −0.72 (−0.71, −0.73) for sphericity, and −0.69 (−0.68, −0.71) for perimeter to surface ratio, with ICC values of 0.83, 0.96, and 0.96, respectively.

**TABLE 4:** Pfirrmann Grade Correlation and Reliability of Individual Features

Feature	Correlation (95% CI)	ICC (95% CI)
Conventional indices		
Peak signal intensity difference	−0.67 (−0.65 –−0.68)	0.60 (0.22–0.78)
IVD height index	−0.45 (−0.43 –−0.47)	0.81 (0.49–0.91)
All features sPLSDA component 1		
Interquartile Range	−0.77 (−0.76 –−0.78)	0.83 (0.07–0.95)
All features sPLSDA component 2		
Sphericity	−0.72 (− 0.71 –−0.73)	0.96 (0.87–0.98)
Perimeter to surface ratio	−0.69 (−0.68 –−0.71)	0.96 (0.86–0.98)
LoG-3 mm GLSZM zone percentage	0.30 (0.28–0.32)	0.97 (0.96–0.98)
LoG-3 mm GLDM SDE	0.17 (0.15–0.20)	0.99 (0.98–0.99)
LoG-3 mm GLCM Idn	−0.12 (−0.10 –−0.15)	0.98 (0.97–0.99)
LoG-3 mm GLRLM run percentage	−0.06 (−0.03 –−0.09)	0.97 (0.13–0.99)
LoG-3 mm GLRLM run length NUN	0.05 (0.02–0.07)	0.95 (0.07–0.99)

The correlation column shows individual feature correlations with Pfirrmann grade using the Spearman correlation coefficient calculated on all deep learning IVD masks (development and test sets combined). ICC column shows ICC values for the features calculated from the deep learning IVD masks *versus* the 283 manually annotated masks. All values have *P* < 0.0001.

CI indicates confidence interval; LoG, Laplacian of Gaussian; NUN, nonuniformity normalized; SDE, small dependence emphasis.

## DISCUSSION

In this study, we identified a robust radiomic signature of IVD degeneration using DL segmentation that can effectively classify Pfirrmann grade and improve on the performance of conventional indices. The improvement is modest (66.0% *vs*. 76.8% BA, 0.55 *vs*. 0.71 Cohen’s kappa), but the radiomic features are more robust to IVD segmentation masks than the conventional indices. The approach also revealed interpretable radiomic features with comparable performance to the full radiomics model (Table 3). We propose individual robust features, 2D sphericity and interquartile range (Figure 2E), as effective alternatives to the conventional indices for IVD degeneration classification. In combination with DL segmentation, these features could facilitate more accurate large-scale association studies to identify specific aspects of IVD degeneration that contribute to pain and disability. If validated, they could also be used to monitor treatment responses and model clinically relevant degeneration trajectories longitudinally.

**Figure 5:**
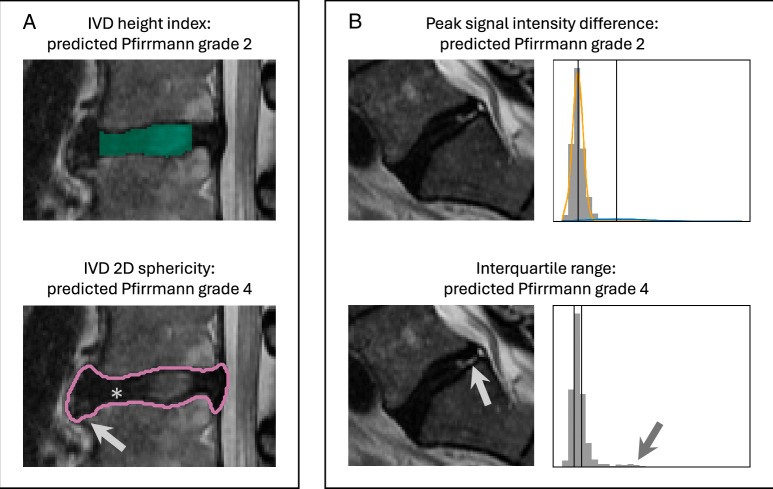
Examples highlighting the differences between the conventional indices and the top two radiomic features. (A) An IVD manually graded as Pfirrmann grade 4 with a high value for IVD height index but a low value for 2D sphericity. This IVD is misclassified as Pfirrmann grade 2 using the conventional indices, and correctly as Pfirrmann grade 4 using the top two radiomics. Osteophytes (gray arrow) and localized loss of disc height (asterisk) impact the 2D sphericity value but not the IVD height index. (B) An IVD manually graded as Pfirrmann grade 4 with high peak signal intensity difference value, but low interquartile range. This IVD is misclassified as Pfirrmann grade 2 using the conventional indices, and correctly as Pfirrmann grade 4 using the top two radiomics. Gray arrows indicate the values for the local areas of high pixel intensity in the image and the pixel intensity histogram. These pixels result in a second “peak” being detected by peak signal intensity difference, whereas the interquartile range is not impacted.

Although the radiomics-based model does not match state-of-the-art DL models trained and tested in the same data set (Kowlagi *et al.* ^40^ had BA of 81.7% and Cohen’s kappa of 0.74) the interpretability of the features is a strength. 2D sphericity may capture a combination of loss of IVD height, osteophyte formation, and vertebral endplate disruption, causing the perimeter to lengthen and the shape of the IVD to flatten. This detects a difference between Pfirrmann grades 2 and 3 while IVD height index does not (Figure 5A). Attempts have been made to incorporate this type of shape change into novel semi-quantitative grading tools,^47^ demonstrating the potential relevance of 2D sphericity to degeneration classification. Interquartile range may improve on peak signal intensity difference by reducing the influence of localized areas of high signal intensity in degenerated IVDs. Nitrogen gas, high intensity zones, and gross morphological changes can cause a U-shaped relationship between many intensity features and Pfirrmann grade,^15^ which is minmized by interquartile range (Figure 5B).

An important strength of our approach is the robustness to DL segmentation. Robustness has not been explored previously for IVD radiomics despite the significant influence it can have on feature calculation and classification accuracy.^48^ The only study we are aware of to examine the reliability of a conventional index from DL segmentations is by Zheng *et al.* ^20^ They compared IVD height index derived from the DL segmentations to a fully manual method, which resulted in an ICC of 0.90 for IVD height index and Spearman’s correlation coefficients with Pfirrmann grade ranging from −0.24 to −0.67. This indicates that even with accurate DL segmentations, the IVD height index is susceptible to significant variation and further underlines the need to select robust features when using DL segmentation.

There are some limitations to our study. Importantly, the radiomic signatures have not been externally validated and are based on a single data set. While the analysis focused on feature robustness to segmentation variations, it does not indicate whether the radiomic signatures can be generalized. Both inter-scanner and intra-scanner (ie, repeat measurement) reliability for IVD radiomics also remain to be established. Other limitations include our use of the numerical mid-sagittal slices, which may not align with the anatomical midline. Although manual screening helped mitigate the issue, future work should incorporate a step to select optimal slices. Accurate identification of IVD levels is also a challenge for DL models, particularly in the presence of transitional vertebrae. Transitional vertebrae have a prevalence of 16% in the NFBC1966, and in this study, over 5% of IVDs were labelled with the incorrect level. It is also worth noting that this study is constrained by training models for Pfirrmann grading. Future work could explore robust texture features from standard-of-care MRI for clusters of IVDs that appear otherwise healthy using an unsupervised approach, with the aim of identifying early subvisual changes indicative of biochemical alterations known to precede visible degeneration.^16^

Our results identify 2D sphericity and interquartile range as robust stand-alone texture features that are highly associated with Pfirrmann grade. These features, if validated, could serve as more reliable alternatives to the conventional indices of IVD height and peak signal intensity difference derived from DL segmentation. Although a more complex radiomic signature also accurately classifies Pfirrmann grade, it does not provide additional information. Overall, the robust radiomic features identified represent a significant advance in the automated quantitative phenotyping of IVD degeneration from standard-of-care MRI. This approach has important implications for the measurement of IVD degeneration severity, monitoring of longitudinal change in the IVD, and prediction of IVD degeneration trajectories or treatment response in large clinical data sets and population cohorts.

Key Points

- Robust radiomic signatures from deep learning segmentation of the intervertebral disc accurately classify Pfirrmann grade with balanced accuracy of 76.7% (73.1%–80.3%) and Cohen’s kappa of 0.70 (0.67–0.74).
- The individual radiomics features, 2D sphericity and interquartile range, alone capture most of the information contained in the radiomic signature and are more effective than disc height index and interquartile range.
- Disc height index and peak signal intensity difference may not be suitable for degeneration classification when derived from deep learning segmentations.
- The combination of deep learning and robust radiomics represents a significant advance in the automated quantitative phenotyping of IVD degeneration from standard-of-care MRI.

## Supplementary Material
