# Supplementary material for: Robust Radiomic Signatures of Intervertebral Disc Degeneration From MRI
Source: Spine (Phila Pa 1976). 2025 Jun 20;50(24):1737–46. doi: 10.1097/BRS.0000000000005435 (PMC12637164; doi:10.1097/BRS.0000000000005435)
Supplement: Supplementary file 1 [file brs-50-1737-s001.pdf]

Supplementary material: Robust radiomic signatures of intervertebral disc degeneration from MRI

Calculation of conventional indices

Intervertebral disc (IVD) height index was calculated using an area-based method [2, 1] as implemented by [4]. The diameters of the two vertebral bodies (VBs) adjacent to each IVD were first determined (Equation 1). The four corners of the VB were identified using the Shi-Tomasi corner detection method. The vertebral body diameter (VD^i) was calculated as the Euclidean distance between the midpoint of the line connecting the two anterior corners ($L^i_{\mathrm{amp},j}$) and the midpoint of the line connecting the two posterior corners ($L^i_{\mathrm{pmp},j}$):

$$\mathrm{VD}^i = \sqrt{\sum_{j=1}^{2}\left(L^i_{\mathrm{amp},j} - L^i_{\mathrm{pmp},j}\right)^2} \tag{1}$$

The vertebral height (VH^i) was calculated as the surface area of the vertebral body divided by its diameter (Equation 2):

$$\mathrm{VH}^i = \frac{1}{\mathrm{VD}^i}\sum_{x=1}^{h}\sum_{y=1}^{w} P_{xy} \tag{2}$$

The IVD height was estimated using an area-based method, following Videman's approach. The IVD area was calculated from the central 80% of the IVD, and the height (DH^i) was obtained by dividing the area by the IVD diameter (Equation 3):

$$\mathrm{DH}^i = \frac{1}{\mu \parallel D^i_a D^i_p \parallel}\sum_{x=\min X_D}^{\max X_D}\sum_{y=\min Y_D}^{\max Y_D} P_{xy} \tag{3}$$

The IVD height was normalized by dividing it by the sum of the heights of the two adjacent vertebrae, as shown in Equation 4:

$$\mathrm{DHI}^i = \frac{2 \times DH^i}{VH^i + VH^{i+1}} \tag{4}$$

The peak signal intensity (SI) difference was calculated using the method of Waldenberg [3], with an additional normalization step (Equation 5). The peak SI was determined by fitting a two-part Gaussian to the disc signal intensity histogram and subtracting the respective Gaussian peak values. Higher values indicated greater contrast between the darker annulus fibrosus (AF) and the lighter nucleus pulposus (NP). This value was normalized by dividing by the mean signal intensity of cerebrospinal fluid (CSF), measured from a circular region of interest (ROI) in the anterior dural sac (3mm diameter), placed as close as possible to the posterior aspect of the disc:

$$\Delta\mathrm{SI}^i = \frac{\mathrm{SI}^i_2 - \mathrm{SI}^i_1}{\mathrm{SI}_{CSF}} \tag{5}$$

Calculation of best 2 radiomic features

Iterquartile range and 2D sphericity were identified as highly relevant radiomic features. These features were calculated using Pyradiomics. *sphericity* is given by:

$$sphericity = \frac{2\pi R}{P} = \frac{2\sqrt{\pi A}}{P} \tag{6}$$

Where R is the radius of a circle with the same surface as the disc mask, and equal to $\sqrt{\frac{A}{\pi}}$, and P is the length of the perimeter of the disc mask. Interquartile range is given by:

$$interquartile\ range = \mathbf{P}_{75} - \mathbf{P}_{25} \tag{7}$$

Where $\mathbf{P}_{25}$ and $\mathbf{P}_{75}$ are the 25th and 75th percentiles of the image array, respectively. All radiomic features other than 2D shape features were calculated on normalised images using the Pyradiomics package. Pyradiomics normalizes the image by centering it at the mean with standard deviation. Normalization uses all gray values in the image.

$$f(x) = \frac{x - \mu_x}{\sigma_x} \tag{8}$$

Where x and $f(x)$ are the original and normalized intensity, respectively, and μ_x and σ_x are the mean and standard deviation of the image intensity values. Using Pyradiomics, outliers in the image with values for which $x > \mu_x + 3\sigma_x$ or $x < \mu_x - 3\sigma_x$ were set to $\mu_x + 3\sigma_x$ and $\mu_x - 3\sigma_x$, respectively.

Identification of and adjustment for disc area dependence

IVD area dependence was checked for all features by fitting linear, logarithmic, exponential, power, and polynomial models to each feature using IVD surface area as the independent variable for all IVDs in the development set. AIC and BIC were used to identify models with the best fit and features with R2 of > 0.4 and p-value of < 0.05 were considered highly area dependent and selected for adjustment. 27 features met these criteria and are listed in Table 1.

Table 1. IVD area dependent radiomic features and best fitting models used for feature adjustment.

Feature	R^2	Model
wavelet-LH_GrayLevelNonUniformity	0.99	power
wavelet-LH_GrayLevelNonUniformity	0.89	power
log-sigma-1-mm_GrayLevelNonUniformity	0.87	power
wavelet-LL_DependenceNonUniformity	0.87	power
wavelet-HL_RunLengthNonUniformity	0.83	power
wavelet-LH_DependenceNonUniformity	0.82	power
MinorAxisLength	0.80	power
log-sigma-5-mm_DependenceNonUniformity	0.79	power
TotalEnergy	0.75	power
log-sigma-1-mm_DependenceNonUniformity	0.72	power
log-sigma-1-mm_Coarseness	0.70	polynomial
log-sigma-3-mm_DependenceNonUniformity	0.70	power
PerimeterSurfaceRatio	0.69	polynomial
wavelet-HH_LargeAreaLowGrayLevelEmphasis	0.69	power
log-sigma-3-mm_LargeAreaHighGrayLevelEmphasis	0.62	power
log-sigma-5-mm_LargeAreaHighGrayLevelEmphasis	0.55	power
wavelet-LH_ZoneVariance	0.53	power
log-sigma-1-mm_GrayLevelNonUniformity	0.51	power
wavelet-HH_ZonePercentage	0.49	polynomial
log-sigma-5-mm_GrayLevelNonUniformity	0.46	power
log-sigma-5-mm_RunEntropy	0.46	power
log-sigma-3-mm_RunEntropy	0.44	power
log-sigma-1-mm_Median	0.43	power
log-sigma-1-mm_LargeAreaHighGrayLevelEmphasis	0.43	power
log-sigma-5-mm_LongRunHighGrayLevelEmphasis	0.41	power
log-sigma-5-mm_ZoneVariance	0.40	power

P-values of < 0.0001 for all listed features.

Features were adjusted in the development set and the test set using the models trained on the development set alone to prevent data leakage. Examples of area dependent features plotted against IVD surface area are shown for the test set in Figure 1. The model predictions pre-adjustment are shown alongside the adjusted features with a new line of best fit.

Feature robustness to image and mask perturbations

ICC2 values were calculated for each feature for each image and mask perturbation against the features calculated from the manually segmented discs. The relative impact on ICC values per perturbation is shown in 2. The feature ICC values across perturbations were clustered to identify a subset of features most robust to the image mask variations (3).

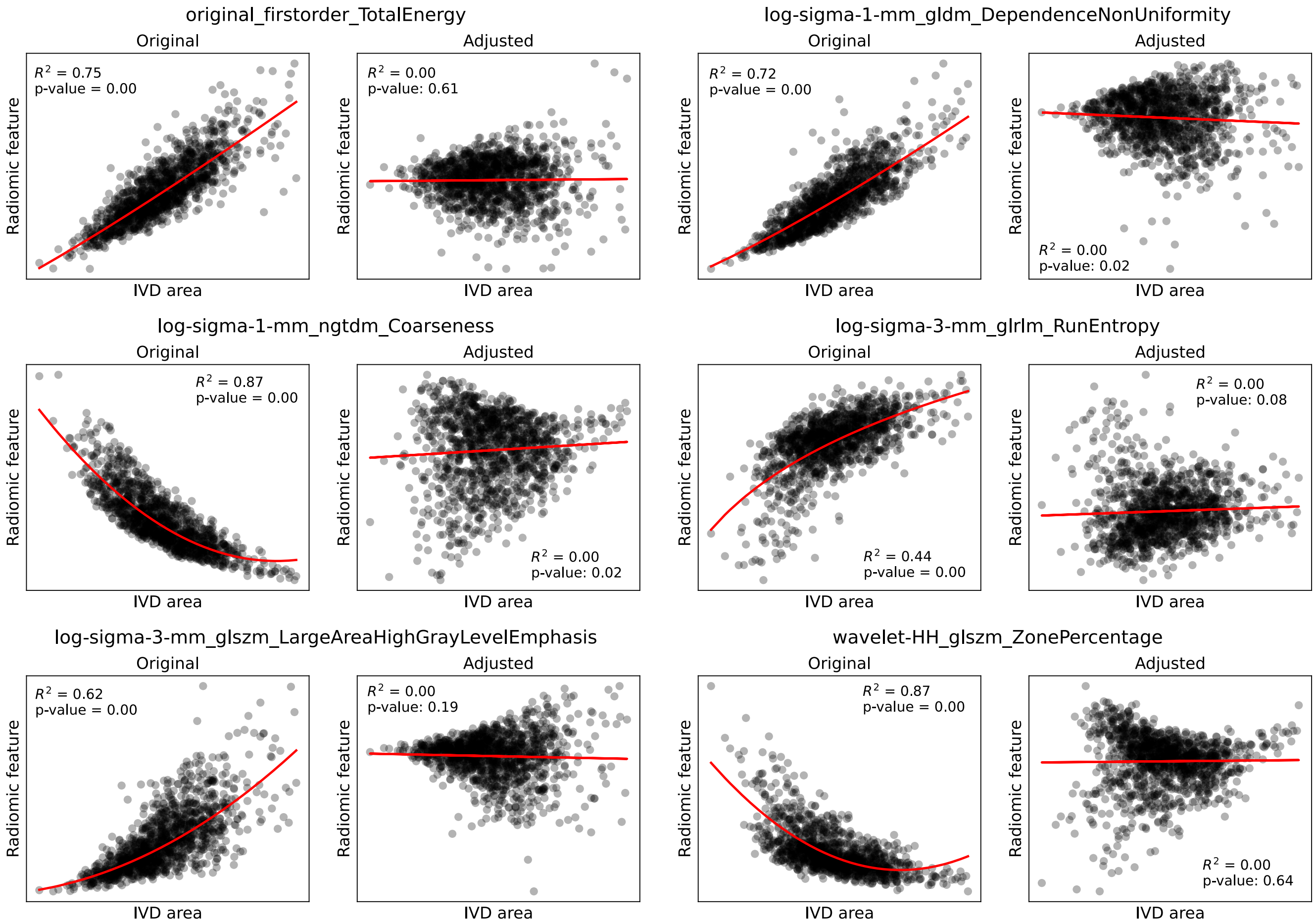

Figure 1. Examples of 6 IVD area dependent radiomic features from the test data plotted against IVD surface area. Panels labelled "original" show the features before area adjustment with a line of best fit in red, panels labelled "adjusted" show the features after adjustment for IVD area with a new line of best fit. The area dependence was adjusted independently in the development and test datasets.

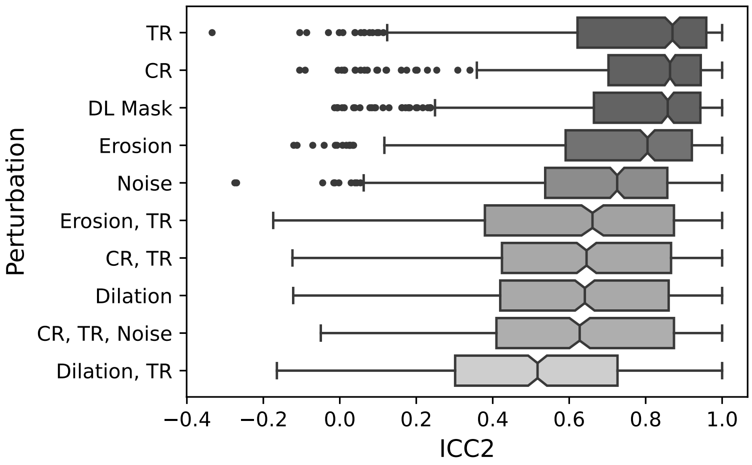

Figure 2. ICC2 values for all features for each image-mask perturbation, in descending order of median value per perturbation. TR: translation, rotation; CR: contour randomisation; DL: deep learning.

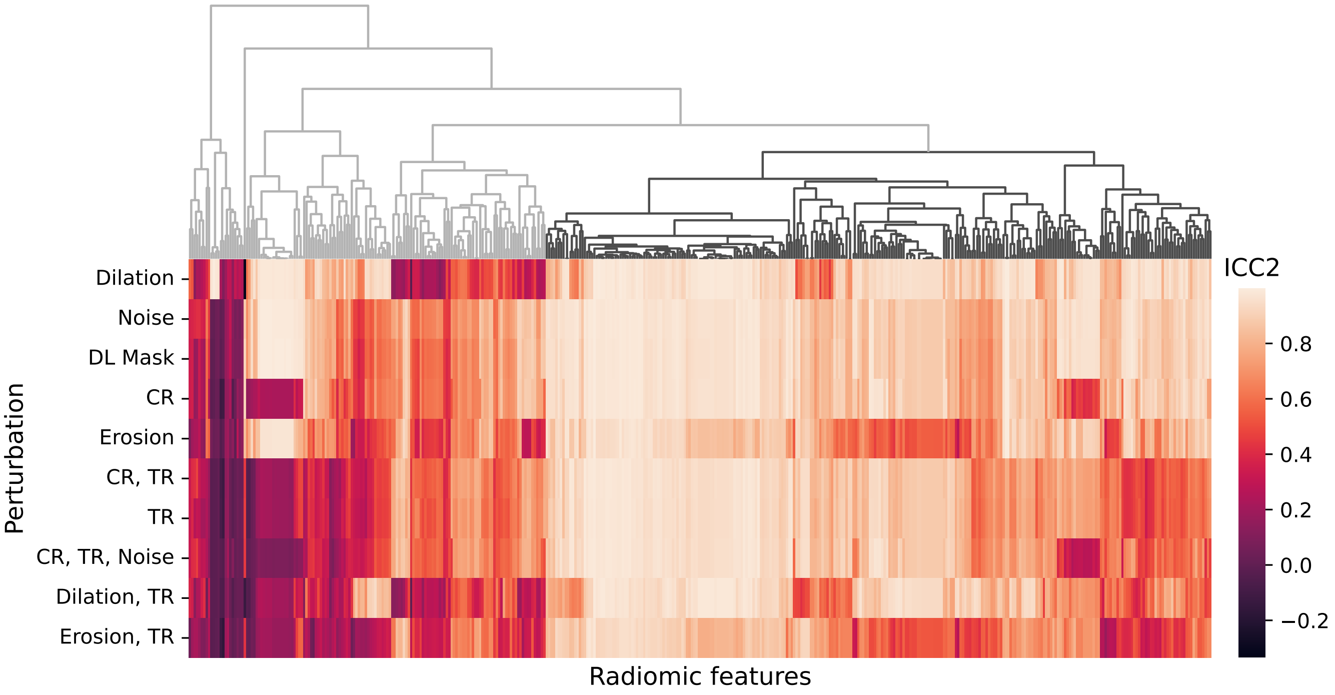

Figure 3. Hierarchically clustered heatmap of ICC2 values for 430 radiomic features (columns) for each image-mask perturbation (rows). The cluster dendrogram of 280 most robust features is shaded in dark gray, and the 4 remaining clusters of less robust features are shaded light gray. TR: translation, rotation; CR: contour randomisation; DL: deep learning.

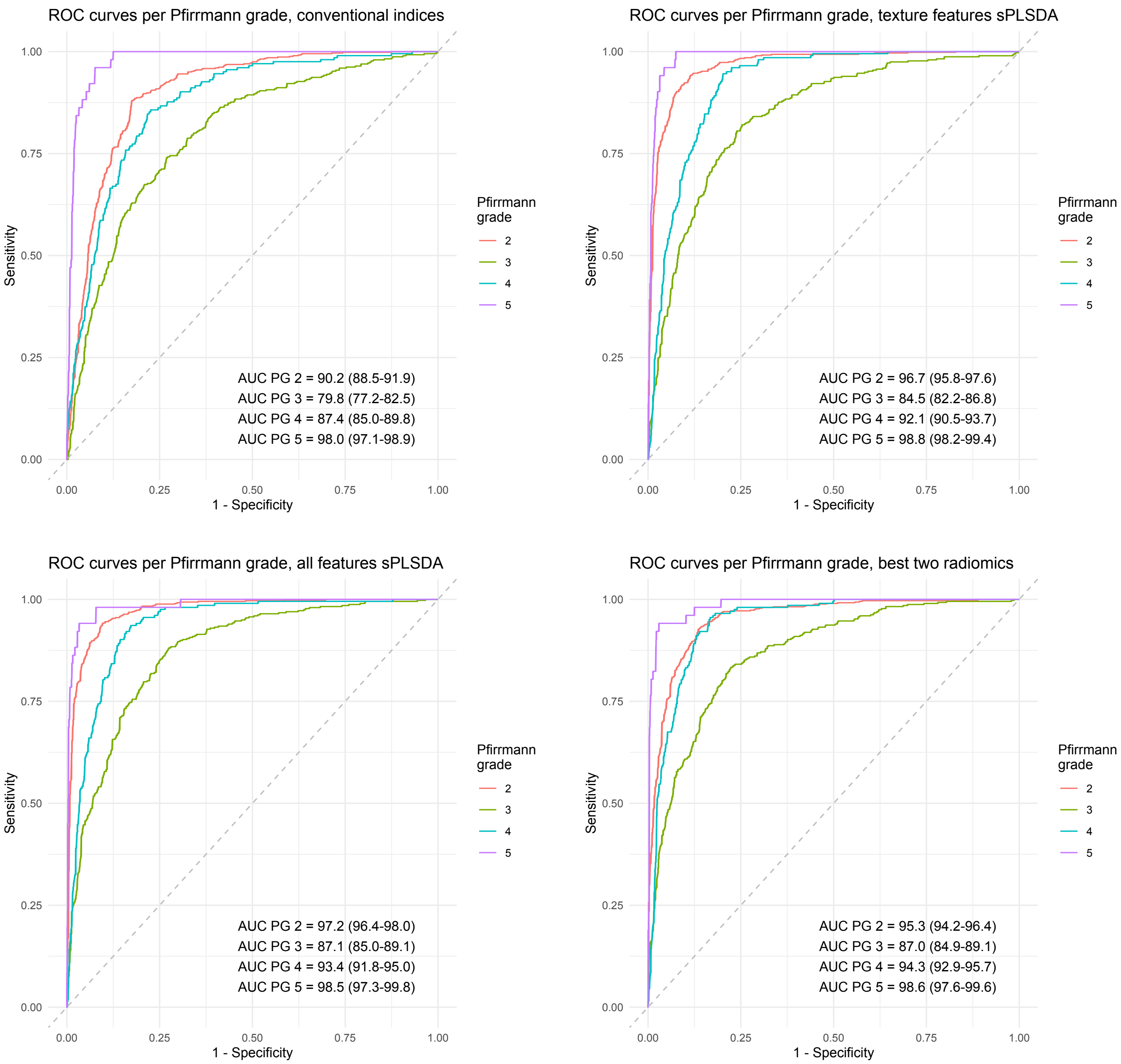

Figure 4. Receiver operator characteristic (ROC) curves per Pfirrgmann grade. AUC: area under curve; PG: Pfirrmann grade.

References

[1] Vahid Abdollah, Eric C. Parent, and Michele C. Battié. "Reliability and validity of lumbar disc height quantification methods using magnetic resonance images". In: *Biomedical Engineering / Biomedizinische Technik* 64.1 (2019), pp. 111–117. ISSN: 0013-5585. DOI: 10.1515/bmt-2017-0086.

[2] Tapio Videman et al. "Progression and Determinants of Quantitative Magnetic Resonance Imaging Measures of Lumbar Disc Degeneration". In: *Spine* 33.13 (2008), pp. 1484–1490. ISSN: 0362-2436. DOI: 10.1097/brs.0b013e3181753bb1.

[3] Christian Waldenberg et al. "MRI histogram analysis enables objective and continuous classification of intervertebral disc degeneration". In: *European Spine Journal* 27.5 (2018), pp. 1042–1048. ISSN: 0940-6719. DOI: 10.1007/s00586-017-5264-7.

[4] Hua-Dong Zheng et al. "Deep learning-based high-accuracy quantitation for lumbar intervertebral disc degeneration from MRI". In: *Nature Communications* 13.1 (2022), p. 841. DOI: 10.1038/s41467-022-28387-5.
